# Supplementary material for: Two distinct regulatory pathways govern Cct2-Atg8 binding in the process of solid aggrephagy
Source: EMBO Rep. 2024 Sep 25;25(11):4749–76. doi: 10.1038/s44319-024-00275-7 (PMC11549370; doi:10.1038/s44319-024-00275-7)
Supplement: Supplementary file 1 — Appendix [file 44319_2024_275_MOESM1_ESM.pdf]

APPENDIX

Two distinct regulatory pathways govern Cct2-Atg8 binding in the process of solid aggrephagy

Yuting Chen#, Zhaojie Liu#, Yi Zhang#, Miao Ye, Yingcong Chen, Jianhua Gao, Juan Song, Huan Yang, Choufei Wu, Weijing Yao, Xue Bai, Mingzhu Fan, Shan Feng, Yigang Wang, Liqin Zhang, Liang Ge, Du Feng*, Cong Yi*

#Co-first author

* Correspondence: Cong Yi, email: yiconglab@zju.edu.cn; Du Feng, email: Fenglab@gzhmu.edu.cn.

Table of Contents

Appendix Figure S1 (Page 2)

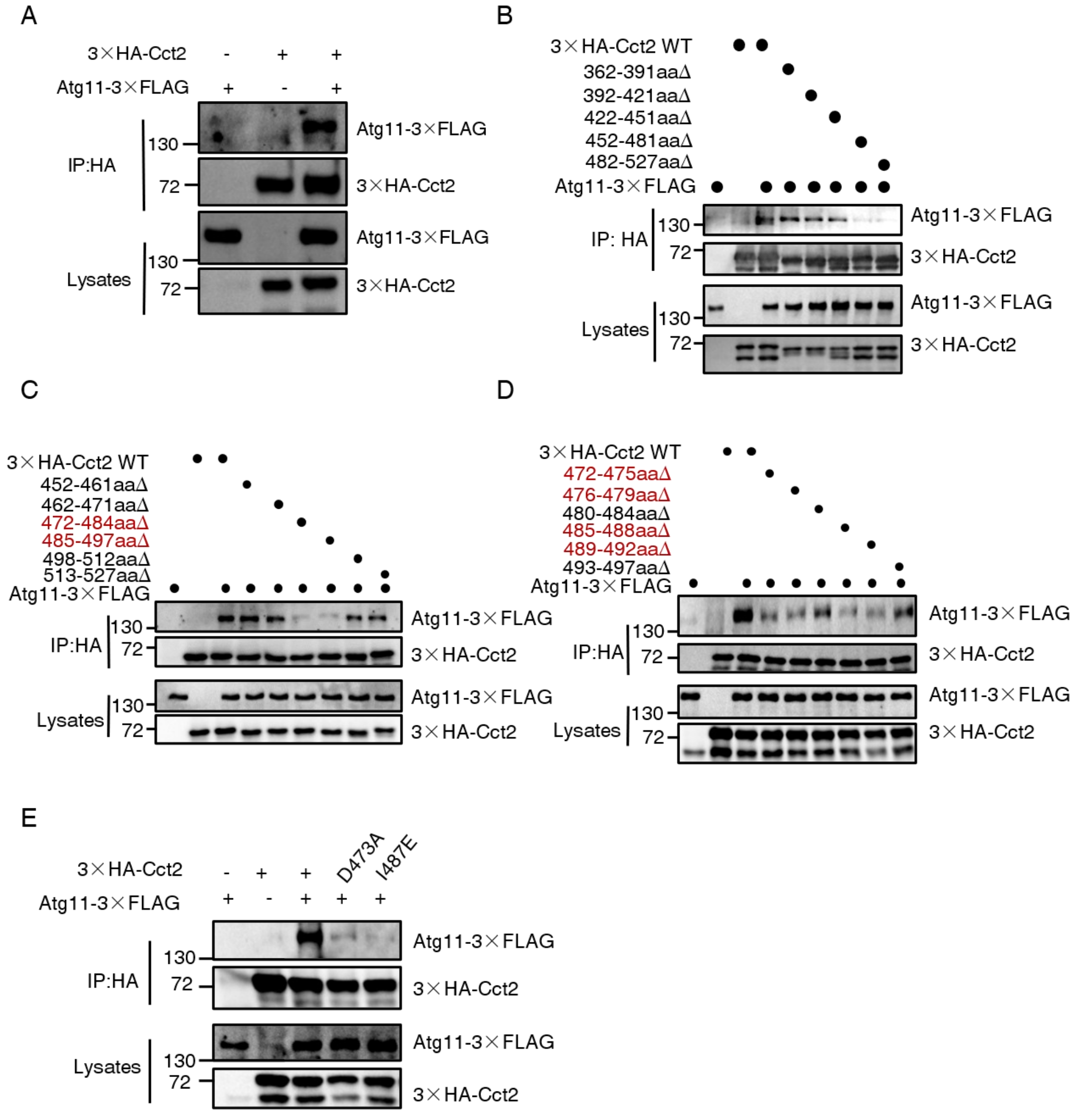

Appendix Figure S1. Identify the key region(s) and amino acid residue(s) on Cct2 that mediate the interaction between Cct2 and Atg11.

(A) Cells co-expressing 3×HA-Cct2 with Atg11-3×FLAG were grown to the log phase. Cell lysates were immunoprecipitated with anti-HA agarose beads and then analyzed by western blot using anti-FLAG antibody. The data are representative of two independent experiments. **(B-E)** Cells co-expressing 3×HA-Cct2 or the indicated Cct2 mutants with Atg11-3×FLAG were grown to the log phase. Cell lysates were immunoprecipitated with anti-HA agarose beads and then analyzed by western blot using anti-FLAG antibody. The data are representative of two independent experiments.
