# Supplementary material for: Two distinct regulatory pathways govern Cct2-Atg8 binding in the process of solid aggrephagy
Source: EMBO Rep. 2024 Sep 25;25(11):4749–76. doi: 10.1038/s44319-024-00275-7 (PMC11549370; doi:10.1038/s44319-024-00275-7)
Supplement: Supplementary file 13 — Expanded View Figures [file 44319_2024_275_MOESM13_ESM.pdf]

Expanded View Figures

Figure EV1. (corresponding to Fig. 1). Cct2 specifically binds to Atg8 and is degraded by autophagy in yeast.

(**A**) BY4741, *cct1-ts*, or *cct4-ts* yeast cells expressing Atg11-3×FLAG were grown to log phase at 30 °C and then subjected to 37 °C for 2 h. Samples were analyzed by immunoblot for detecting the expression of Atg11-3×FLAG and α-Tubulin. Pgk1 served as a loading control. The data are representative of three independent experiments. (**B**) GST pulldowns were performed by using purified GST, GST-Cct1, GST-Cct2, or GST-Cct4 with His-Atg8 protein from *E. Coli*. The data are representative of three independent experiments. (**C**) *ape1Δ* cells co-expressing HA-Cct1, HA-Cct2, HA-Cct4, HA-Cct5, or HA-Cct8 with FLAG-Atg8 and Ape1 P22L were grown to the log phase under nutrient-rich conditions. Cell lysates were immunoprecipitated with anti-FLAG agarose beads and then analyzed by western blot using anti-HA antibody. The data are representative of three independent experiments. (**D**) *ape1Δ*, *ape1Δ atg8Δ*, *ape1Δ atg11Δ*, *ape1Δ atg15Δ*, *ape1Δ atg2Δ atg15Δ*, or *ape1Δ pep4Δ* cells co-expressing Cct2-GFP and Vph1-mCherry in the presence of Ape1 P22L were grown to $OD_{600} = 0.8$. These yeast strains were then cultured in nutrient-rich medium for 6 h. Images of cells were obtained using an inverted fluorescence microscope. Scale bar, 2 μm. (**E**) Cells from (**D**) were quantified for the vacuolar localization of Cct2-GFP. $n = 300$ cells were pooled from three independent experiments. Data are presented as means ± SD. ***$P < 0.001$; two-tailed Student's t tests were used. $P < 0.0001$(*atg8Δ* vs. WT); $P < 0.0001$(*atg11Δ* vs. WT); $P < 0.0001$(*atg15Δ* vs. WT); $P < 0.0001$(*atg2Δ*& *atg15Δ* vs. WT); $P < 0.0001$(*pep4Δ* vs. WT) in (**E**).

© The Author(s)

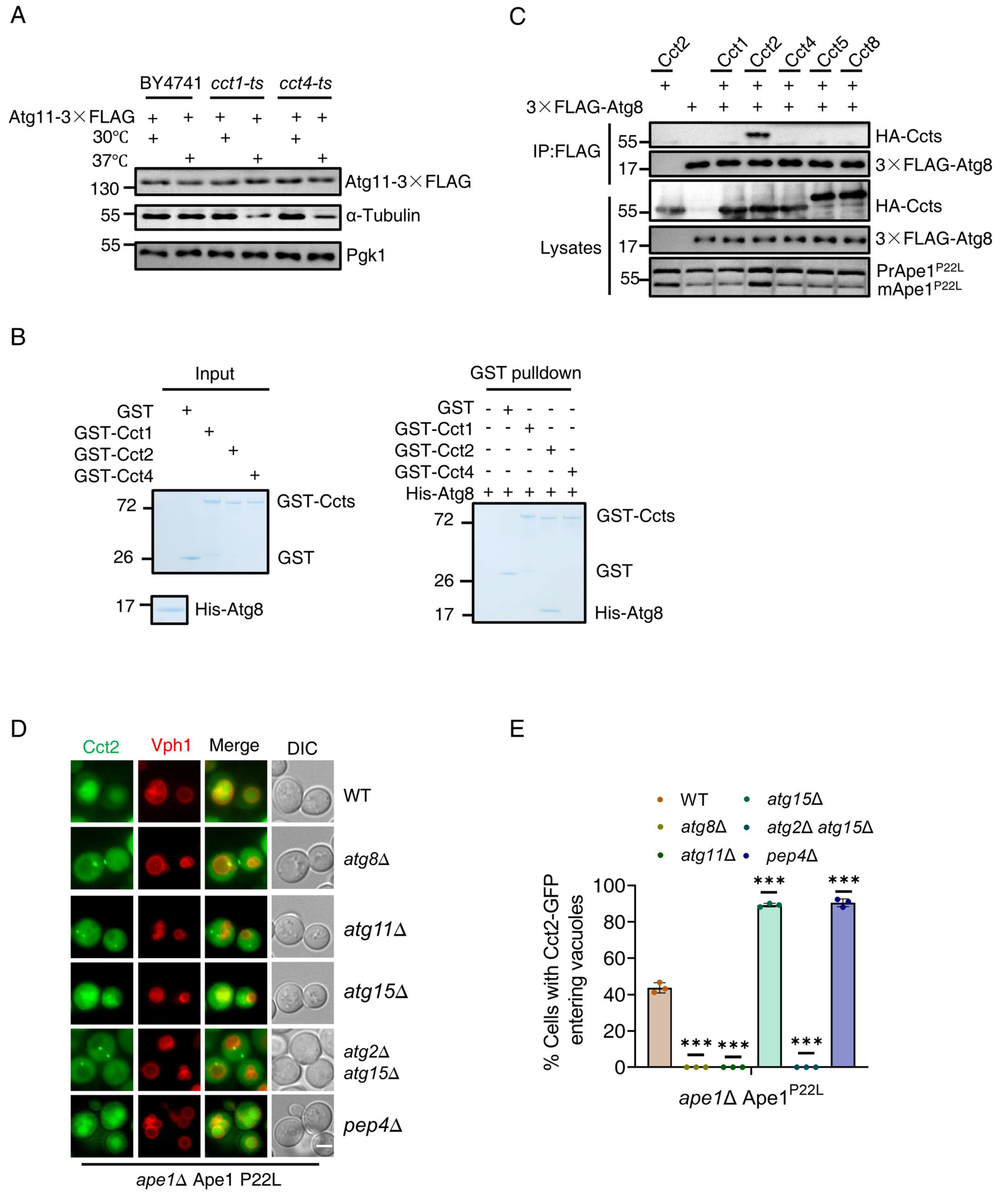
A
BY4741
cct1-ts
cct4-ts
Atg11-3×FLAG
30℃
37℃
130
55
55
Atg11-3×FLAG
α-Tubulin
Pgk1
B
Input
GST
GST-Cct1
GST-Cct2
GST-Cct4
72
26
17
GST-Ccts
GST
His-Atg8
GST pulldown
GST
GST-Cct1
GST-Cct2
GST-Cct4
His-Atg8
72
26
17
GST-Ccts
GST
His-Atg8
C
Cct2
Cct1
Cct2
Cct4
Cct5
Cct8
3×FLAG-Atg8
IP:FLAG
Lysates
55
17
55
17
55
HA-Ccts
3×FLAG-Atg8
HA-Ccts
3×FLAG-Atg8
PrApe1P22L
mApe1P22L
D
Cct2
Vph1
Merge
DIC
WT
atg8Δ
atg11Δ
atg15Δ
atg2Δ atg15Δ
pep4Δ
ape1Δ Ape1 P22L
E
WT
atg8Δ
atg11Δ
atg15Δ
atg2Δ atg15Δ
pep4Δ
% Cells with Cct2-GFP entering vacuoles
100
80
60
40
20
0
***
ape1Δ Ape1P22L

 © The Author(s)

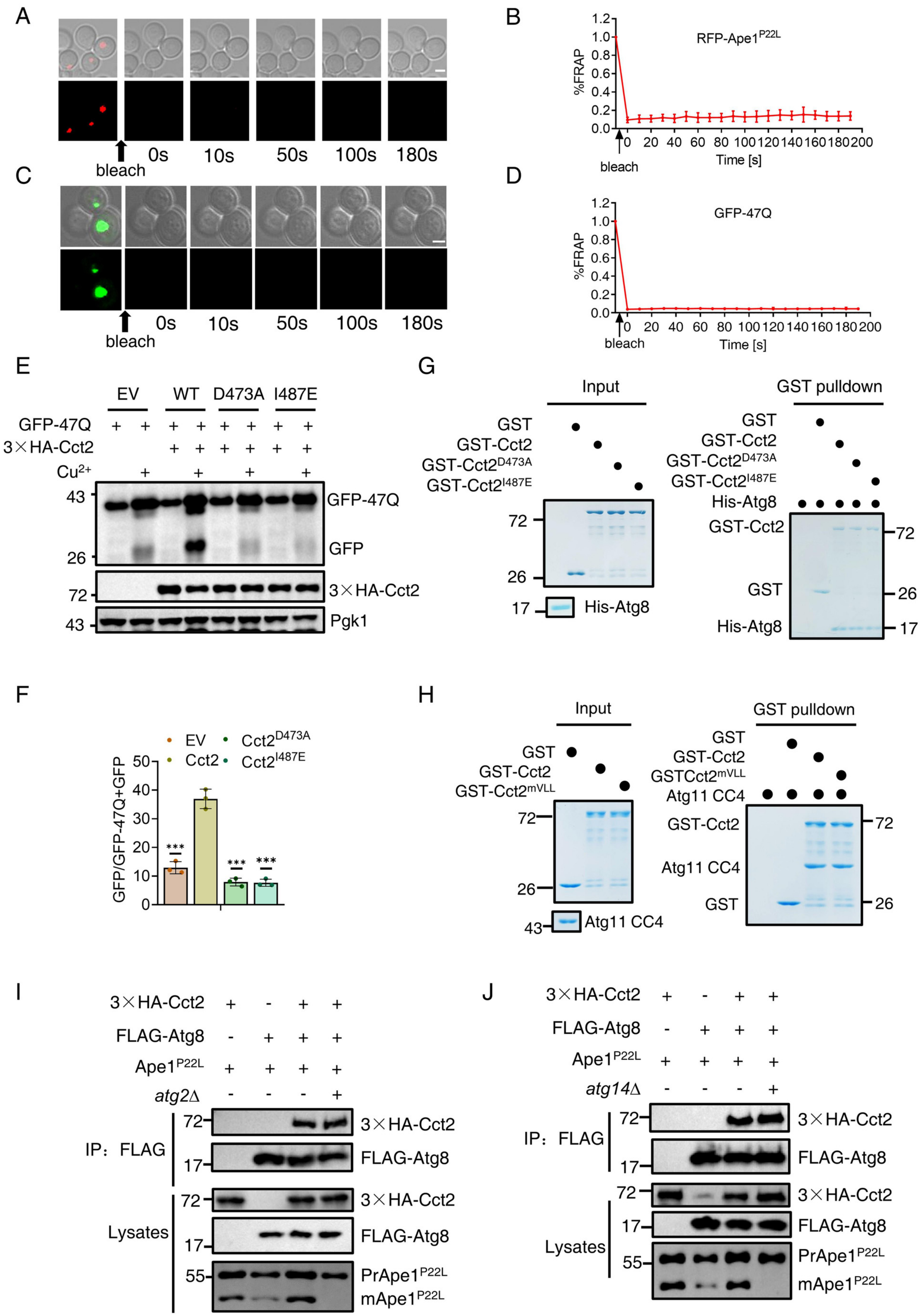
A
bleach
0s
10s
50s
100s
180s
B
RFP-Ape1P22L
%FRAP
Time [s]
bleach
C
D
GFP-47Q
E
EV
WT
D473A
I487E
GFP-47Q
3×HA-Cct2
Cu2+
GFP
Pgk1
F
EV
Cct2
Cct2D473A
Cct2I487E
GFP/GFP-47Q+GFP
***
G
Input
GST pulldown
GST
GST-Cct2
GST-Cct2D473A
GST-Cct2I487E
His-Atg8
H
GST-Cct2mVLL
GSTCct2mVLL
Atg11 CC4
I
FLAG-Atg8
Ape1P22L
atg2Δ
IP: FLAG
Lysates
PrApe1P22L
mApe1P22L
J
atg14Δ

Figure EV2. (corresponding to Fig. 3). The binding of Cct2 to Atg11 is required for solid aggrephagy.

(**A**) *ape1Δ* yeast cells expressing RFP-Ape1 P22L were grown to log phase. FRAP analysis was performed at the indicated time points. Scale bar, 2 µm. (**B**) Quantification of the normalized RFP-Ape1 P22L fluorescence signal (mean ± SD) in (**A**) (>30 cells from three independent experiments. (**C**) BY4741 yeast cells expressing Cu^{2+}-inducible GFP-47Q plasmids were cultured in nutrient-rich medium for 12 h to induce the formation of solid aggregates. FRAP analysis was performed at the indicated time points. Scale bar: 2 µm. (**D**) Quantification of the normalized GFP-47Q fluorescence signal (mean ± SD) in (**C**) (>30 cells from three independent experiments). (**E**) Yeast cells co-expressing Cu^{2+}-inducible GFP-47Q plasmids with an empty vector (EV), 3×HA-Cct2, or 3×HA-Cct2 variants were grown to an $OD_{600} = 0.6$. Subsequently, 0.1 mM $CuSO_4$ was added to the cells to induce GFP-47Q to form solid aggregates for 12 h. Samples were analyzed by immunoblot for the cleavage of GFP-47Q. Pgk1 served as a loading control. (**F**) Quantification of GFP to GFP-47Q + GFP ratio from (**E**). Data are presented as means ± SD (Data represent the results of three independent experiments). ***$P < 0.001$; two-tailed Student's *t* tests were used. $P = 0.0005$ (EV vs. Cct2); $P = 0.0002$ (D473A vs. Cct2); $P = 0.0002$ (I487E vs. Cct2). (**G**) In vitro GST pulldowns were performed using His-Atg8 with GST, GST-Cct2, GST-Cct2 D473A, or I487E purified from *E. coli*. Protein samples were separated by SDS-PAGE and detected using Coomassie blue staining. The data are representative of two independent experiments. (**H**) In vitro GST pulldowns were performed using Atg11 CC4 with GST, GST-Cct2, GST-Cct2 mVLL (AIM motif mutant) purified from *E. coli*. Protein samples were separated by SDS-PAGE and detected using Coomassie blue staining. The data are representative of two independent experiments. (**I**, **J**) *ape1Δ*, *atg2Δ ape1Δ*, or *atg14Δ ape1Δ* cells co-expressing 3×HA-Cct2 with FLAG-Atg8 in the presence of Ape1 P22L were grown to the log phase under nutrient-rich conditions. Cell lysates were immunoprecipitated with anti-FLAG agarose beads and then analyzed by western blot using anti-HA antibody. The data are representative of three independent experiments.

 © The Author(s)

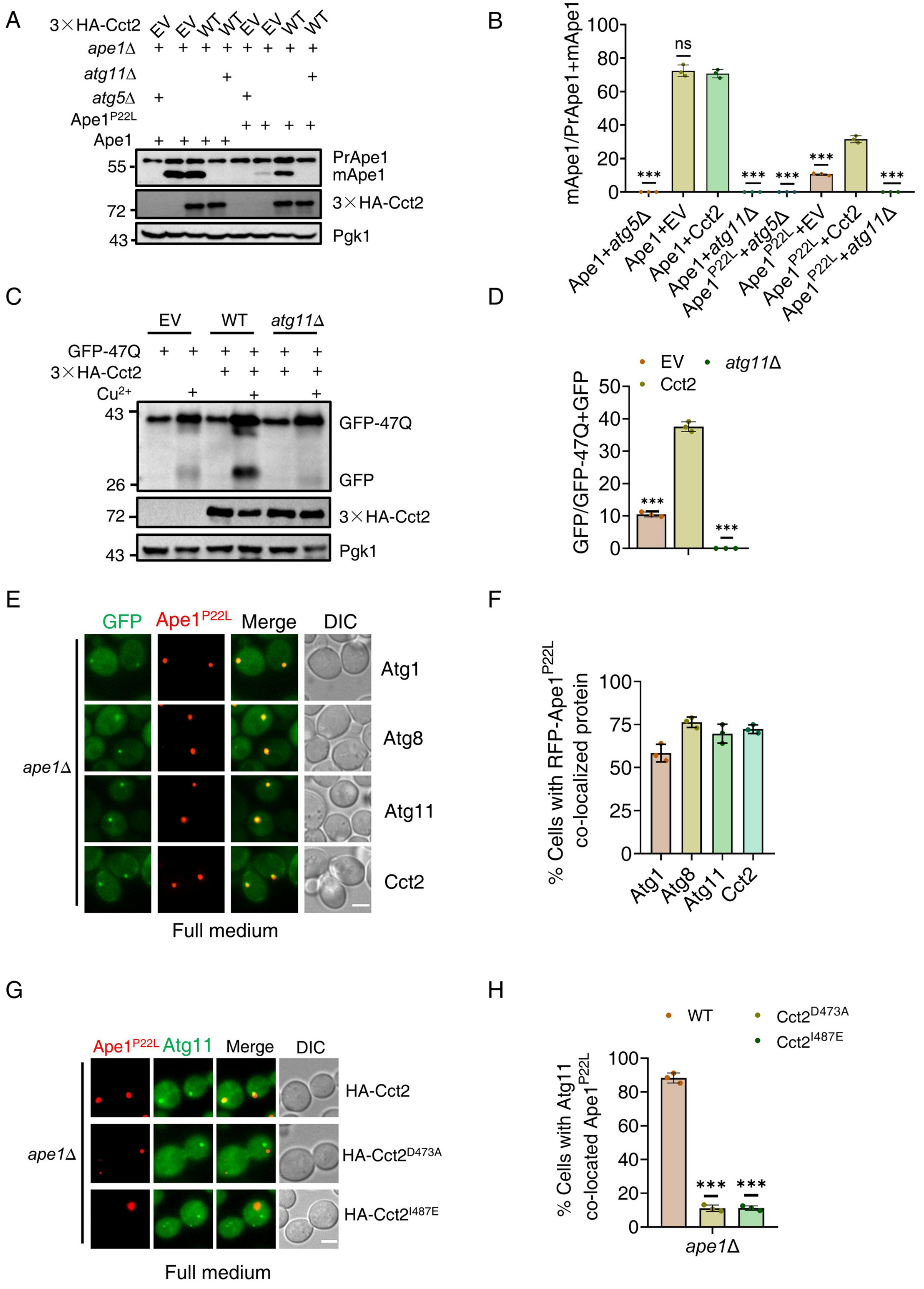
A
3×HA-Cct2 EV EV WT WT EV EV WT WT
ape1Δ + + + + + + + +
atg11Δ + +
atg5Δ + +
Ape1P22L + + + +
Ape1 + + + +
55
72
43
PrApe1
mApe1
3×HA-Cct2
Pgk1
B
mApe1/PrApe1+mApe1
100
80
60
40
20
0
ns
***
Ape1+atg5Δ
Ape1+EV
Ape1+Cct2
Ape1+atg11Δ
Ape1P22L+atg5Δ
Ape1P22L+EV
Ape1P22L+Cct2
Ape1P22L+atg11Δ
C
EV WT atg11Δ
GFP-47Q + + + + + +
3×HA-Cct2 + + + +
Cu2+ + + +
43
26
72
43
GFP-47Q
GFP
3×HA-Cct2
Pgk1
D
EV atg11Δ
Cct2
GFP/GFP-47Q+GFP
50
40
30
20
10
0
***
E
GFP Ape1P22L Merge DIC
Atg1
Atg8
Atg11
Cct2
ape1Δ
Full medium
F
% Cells with RFP-Ape1P22L co-localized protein
100
75
50
25
0
Atg1
Atg8
Atg11
Cct2
G
Ape1P22L Atg11 Merge DIC
HA-Cct2
HA-Cct2D473A
HA-Cct2I487E
ape1Δ
Full medium
H
WT
Cct2D473A
Cct2I487E
% Cells with Atg11 co-located Ape1P22L
100
80
60
40
20
0
***
ape1Δ

Figure EV3. (corresponding to Fig. 3). Atg11 is required for aggregates turnover and Ape1 P22L co-localizes with autophagy-related proteins and Cct2.

(**A**) The indicated yeast cells were grown to the log phase in nutrient-rich medium. Samples were analyzed by immunoblot for detecting the maturation of PrApe1 into mApe1. Pgk1 served as a loading control. (**B**) Quantification of mApe1 to PrApe1+mApe1 ratio from (**A**). Data are presented as means ± SD (Data represent the results of three independent experiments). ***$P < 0.001$; NS, not significant; two-tailed Student's *t* tests were used. $P < 0.0001$ (WT *atg5*Δ vs. WT Cct2); $P = 0.5412$ (WT EV vs. WT Cct2); $P < 0.0001$ (WT *atg11*Δ vs. WT Cct2); $P < 0.0001$ (P22L *atg5*Δ vs. P22L Cct2); $P < 0.0001$ (P22L EV vs. P22L Cct2); $P < 0.0001$ (P22L *atg11*Δ vs. P22L Cct2). (**C**) The indicated yeast cells were grown to an $OD_{600} = 0.6$. Subsequently, 0.1 mM $CuSO_4$ was added to the cells to induce GFP-47Q to form solid aggregates for 12 h. Samples were analyzed by immunoblot for the cleavage of GFP-47Q. Pgk1 served as a loading control. (**D**) Quantification of GFP to GFP-47Q + GFP ratio from (**C**). Data are presented as means ± SD (Data represent the results of three independent experiments). ***$P < 0.001$; two-tailed Student's *t* tests were used. $P < 0.0001$ (EV vs. Cct2); $P < 0.0001$ (*atg11*Δ vs. Cct2). (**E**) *ape1*Δ yeast cells co-expressing RFP-Ape1 P22L with Atg1-2×GFP, GFP-Atg8, Atg11-2×GFP, or Cct2-GFP were grown to the log phase in nutrient-rich medium. Images of cells were obtained using an inverted fluorescence microscope. Scale bar, 2 μm. (**F**) Cells from (**E**) were quantified for the number of cells in which RFP-Ape1 P22L colocalized with the indicated GFP-fused proteins. $n = 300$ cells were pooled from three independent experiments. Data are shown as mean ± SD. (**G**) *ape1*Δ yeast cells co-expressing RFP-Ape1 P22L and Atg11-2×GFP with 3×HA-Cct2, 3×HA-Cct2 D473A, or 3×HA-Cct2 I487E were grown to the log phase in nutrient-rich medium. Images of cells were obtained using an inverted fluorescence microscope. Scale bar, 2 μm. (**H**) Cells from (**G**) were quantified for the number of cells in which RFP-Ape1 P22L colocalized with Atg11-2×GFP. $n = 300$ cells were pooled from three independent experiments. Data are shown as mean ± SD. ***$P < 0.001$; two-tailed Student's *t* tests were used. $P < 0.0001$ (D473A vs. WT); $P < 0.0001$ (I487E vs. WT).

 © The Author(s)

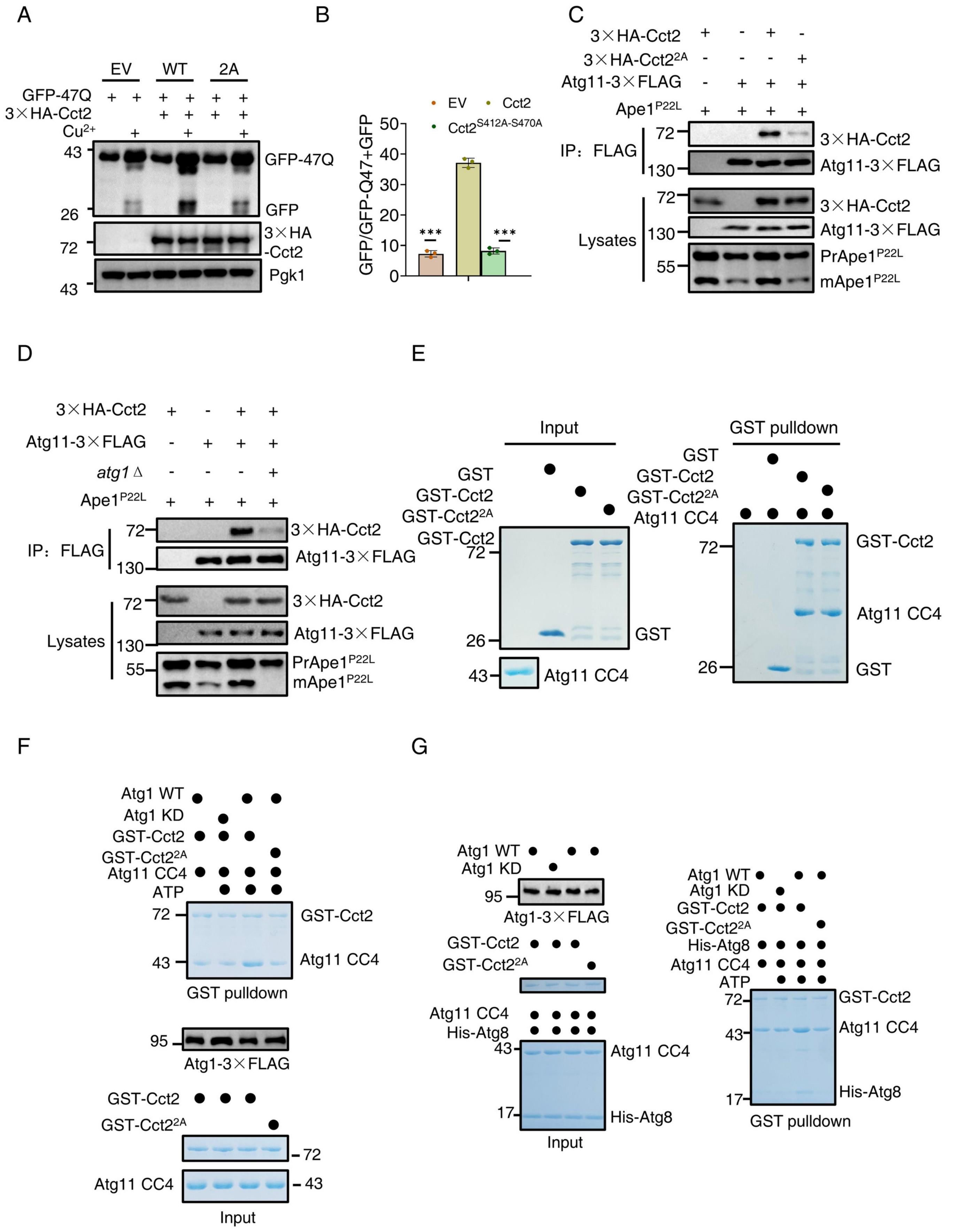
A
EV WT 2A
GFP-47Q
3×HA-Cct2
Cu2+
43
26
72
43
GFP-47Q
GFP
3×HA
-Cct2
Pgk1
B
EV
Cct2
Cct2S412A-S470A
GFP/GFP-Q47+GFP
50
40
30
20
10
0
***
***
C
3×HA-Cct2
3×HA-Cct22A
Atg11-3×FLAG
Ape1P22L
IP: FLAG
Lysates
72
130
55
3×HA-Cct2
Atg11-3×FLAG
PrApe1P22L
mApe1P22L
D
3×HA-Cct2
Atg11-3×FLAG
atg1Δ
Ape1P22L
IP: FLAG
Lysates
72
130
55
3×HA-Cct2
Atg11-3×FLAG
PrApe1P22L
mApe1P22L
E
Input
GST pulldown
GST
GST-Cct2
GST-Cct22A
Atg11 CC4
72
26
43
GST-Cct2
GST
Atg11 CC4
F
Atg1 WT
Atg1 KD
GST-Cct2
GST-Cct22A
Atg11 CC4
ATP
72
43
GST-Cct2
Atg11 CC4
GST pulldown
95
Atg1-3×FLAG
Input
G
Atg1 WT
Atg1 KD
95
Atg1-3×FLAG
GST-Cct2
GST-Cct22A
Atg11 CC4
His-Atg8
43
17
Input
ATP
72
GST pulldown

Figure EV4. (corresponding to Fig. 5). The phosphorylation of Cct2 by Atg1 is required for the cleavage of GFP-47Q and the binding of Cct2-Atg11.

(**A**) Yeast cells co-expressing Cu^{2+}-inducible GFP-47Q plasmids with an empty vector (EV), 3×HA-Cct2, or Cct2 $^{S412A-S470A}$ (2A) were grown to an $OD_{600} = 0.6$. Subsequently, 0.1 mM $CuSO_4$ was added to the cells to induce GFP-47Q to form solid aggregates over 12 h. Samples were analyzed by immunoblot for the cleavage of GFP-47Q. Pgk1 served as a loading control. (**B**) Quantification of GFP to GFP-47Q + GFP ratio from (**A**). Data are presented as means ± SD (Data represent the results of three independent experiments). ***$P < 0.001$; two-tailed Student's *t* tests were used. $P < 0.0001$ (EV vs. Cct2); $P < 0.0001$ (S412A-S470A vs. Cct2). (**C**) *ape1*Δ cells co-expressing an empty vector, 3×HA-Cct2, or 3×HA-Cct2 $^{S412A-S470A}$ with Atg11-3×FLAG in the presence of Ape1 P22L were grown to the log phase under nutrient-rich medium. Cell lysates were immunoprecipitated with anti-FLAG agarose beads and then analyzed by western blot using anti-HA antibody. The data are representative of three independent experiments. (**D**) *ape1*Δ or *ape1*Δ *atg1*Δ cells co-expressing 3×HA-Cct2 with Atg11-3×FLAG in the presence of Ape1 P22L were grown to the log phase under nutrient-rich conditions. Cell lysates were immunoprecipitated with anti-FLAG agarose beads and then analyzed by western blot using anti-HA antibody. The data are representative of three independent experiments. (**E**) In vitro GST pulldowns were performed using Atg11 CC4 domain with GST, GST-Cct2, or GST-Cct2 $^{S412A-S470A}$ purified from *E. coli*. Protein samples were separated by SDS-PAGE and detected using Coomassie blue staining. The data are representative of two independent experiments. (**F**) In vitro phosphorylation assays were performed using GST-Cct2 WT or 2A(S412A-S470A) purified from *E. coli* as substrates, with WT or KD Atg1-3×FLAG, purified from yeast cells, in the presence of Ape1 P22L under nutrient-rich conditions as the protein kinase. After that, in vitro GST pulldowns were performed using phosphorylated products enriched by GST beads in vitro with Atg11 CC4 protein purified from *E. coli*. Protein samples were separated by SDS-PAGE, and then detected using Coomassie blue staining. The data are representative of three independent experiments. (**G**) In vitro phosphorylation assays were performed using GST-Cct2 WT or 2A(S412A-S470A) purified from *E. coli* as substrates, with WT or KD Atg1-3×FLAG, purified from yeast cells, in the presence of Ape1 P22L under nutrient-rich conditions as the protein kinase. After that, in vitro GST pulldowns were performed using phosphorylated products enriched by GST beads in vitro with the same amount of His-Atg8 protein and Atg11 CC4 protein purified from *E. coli*. Protein samples were separated by SDS-PAGE, and then detected using Coomassie blue staining. The data are representative of three independent experiments.

 © The Author(s)

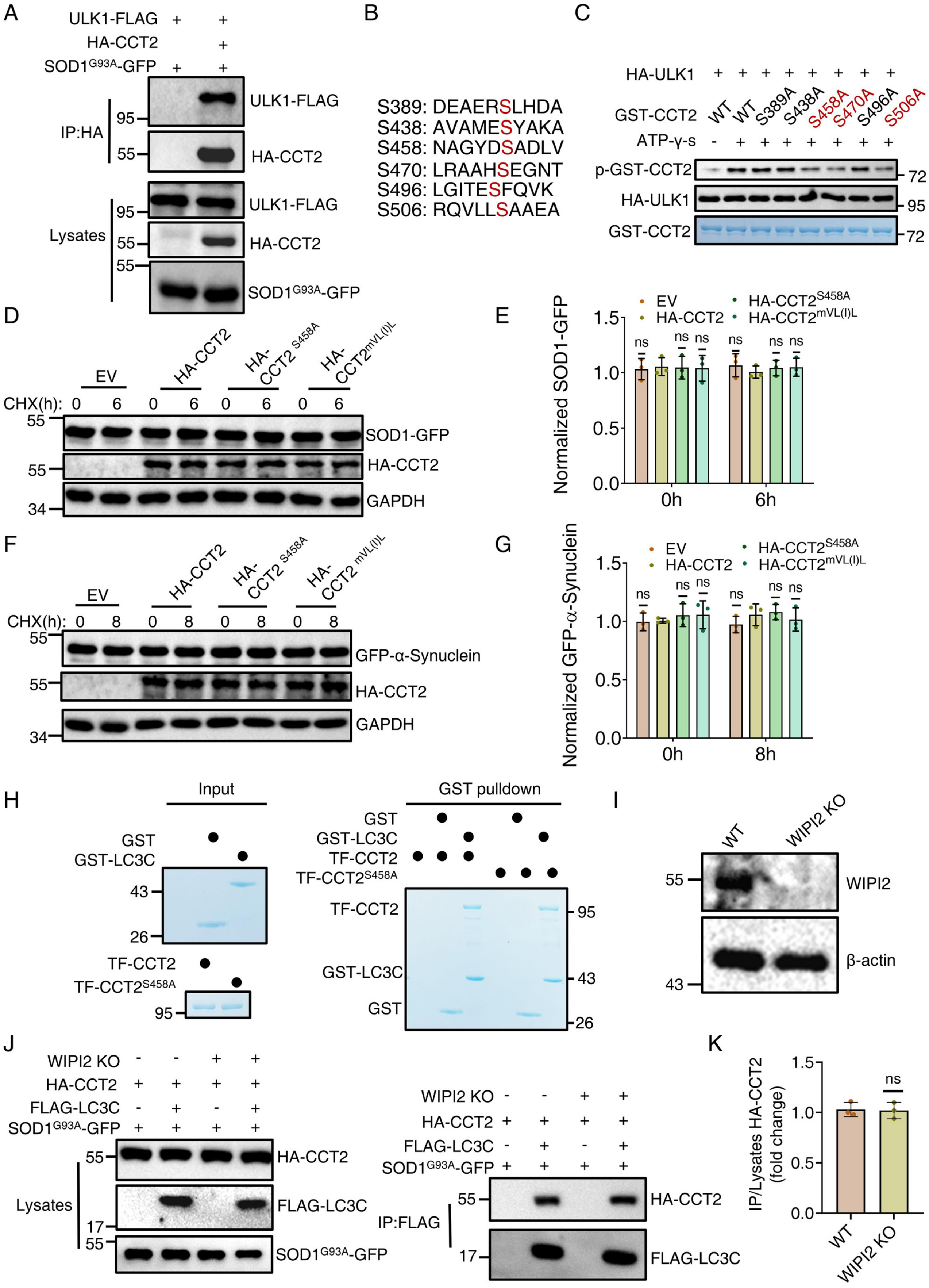
A
ULK1-FLAG
HA-CCT2
SOD1G93A-GFP
IP:HA
Lysates
B
S389: DEAERSLHDA
S438: AVAMESYAKA
S458: NAGYDSADLV
S470: LRAAHSEGNT
S496: LGITESFQVK
S506: RQVLLSAAEA
C
HA-ULK1
GST-CCT2
WT WT S389A S438A S458A S470A S496A S506A
ATP-γ-s
p-GST-CCT2
D
EV HA-CCT2 HA-CCT2 S458A HA-CCT2mVL(I)L
CHX(h):
SOD1-GFP
GAPDH
E
Normalized SOD1-GFP
0h 6h
F
GFP-α-Synuclein
G
Normalized GFP-α-Synuclein
0h 8h
H
Input
GST pulldown
GST
GST-LC3C
TF-CCT2
TF-CCT2S458A
I
WT WIPI2 KO
WIPI2
β-actin
J
WIPI2 KO
FLAG-LC3C
IP:FLAG
K
IP/Lysates HA-CCT2 (fold change)
ns

Figure EV5. (corresponding to Fig. 6). CCT2 is a substrate of ULK1 and WIPI2 does not participate in the binding of LC3C-CCT2 in mammalian cells.

(**A**) HEK293T cell lines were co-transfected with HA-CCT2, ULK1-FLAG, and SOD1 G93A-GFP. Cell lysates were immunoprecipitated with anti-HA agarose beads and then analyzed by western blot using an anti-FLAG antibody. The data are representative of three independent experiments. (**B**) Six typical ULK1 phosphorylation sites on GST-CCT2 D3 domain. (**C**) An in vitro kinase assays were performed using GST, GST-hCCT2, or six indicated GST-hCCT2 variants was purified from *E. coli* as substrates and purified HA-ULK1 from SOD1-G93A-GFP expressed HEK293T cell in nutrient-rich medium as protein kinase. The phosphorylation of Cct2 and these variants were detected by anti-thioP antibody. The data are representative of two independent experiments. (**D**) Turnover of SOD1-GFP in CHX chase assay with empty vector (EV), HA-CCT2, HA-CCT2 ^{S458A}, or HA-CCT2-mVL(I)L (LIR motif mutant) expression in HeLa cell lines at 24 h after transfection. The protein sample were analyzed by western blot. GAPDH served as a loading control. (**E**) Quantification of normalized SOD1-GFP from (**D**). Data are presented as means ± SD (Data represent the results of three independent experiments). NS, not significant. Two-tailed Student's *t* tests were used. $P = 0.7644$ (EV 0 h vs. CCT2 0 h); $P = 0.9012$ (S458A 0 h vs. CCT2 0 h); $P = 0.8480$ (mVL(I)L 0 h vs. CCT2 0 h); $P = 0.4273$ (EV 6 h vs. CCT2 6 h); $P = 0.5161$ (S458A 6 h vs. CCT2 6 h); $P = 0.4892$ (mVL(I)L 6 h vs. CCT2 6 h) (**F**) Turnover of GFP-α-Synuclein in CHX chase assay with empty vector (EV), HA-CCT2, HA-CCT2 ^{S458A}, or HA-CCT2-mVL(I)L (LIR motif mutant) expression in HeLa cell lines at 24 h after transfection. The protein sample were analyzed by western blot. GAPDH served as a loading control. (**G**) Quantification of normalized GFP-α-Synuclein (**F**). Data are presented as means ± SD (Data represent the results of three independent experiments). NS, not significant. Two-tailed Student's *t* tests were used. $P = 0.8375$ (EV 0 h vs. CCT2 0 h); $P = 0.4615$ (S458A 0 h vs. CCT2 0 h); $P = 0.5141$ (mVL(I)L 0 h vs. CCT2 0 h); $P = 0.2845$ (EV 8 h vs. CCT2 8 h); $P = 0.7403$ (S458A 8 h vs. CCT2 8 h); $P = 0.6388$ (mVL(I)L 8 h vs. CCT2 8 h). (**H**) In vitro GST pulldowns were performed using GST-LC3C domain with TF-hCCT2 or TF-hCCT2^{S458A} purified from *E. coli*. Protein samples were separated by SDS-PAGE and detected using Coomassie blue staining. The data are representative of two independent experiments. (**I**) Western blot validation of WIPI2 KO effect in Hela cell lines. The data are representative of two independent experiments. (**J**) WT or WIPI2 KO Hela cell lines were co-transfected with FLAG-LC3C, HA-CCT2, and SOD1 G93A-GFP. Cell lysates were immunoprecipitated with anti-FLAG agarose beads and then analyzed by western blot using an anti-HA antibody. (**K**) Quantification of IP/lysates HA-CCT2 from (**J**). Data are presented as means ± SD (Data represent the results of three independent experiments). NS, not significant; two-tailed Student's *t* tests were used. $P = 0.8785$ (WIPI2 KO vs. WT).

© The Author(s)
